# Supplementary material for: Treatment burden in individuals living with and beyond cancer: A systematic review of qualitative literature
Source: PLoS One. 2023 May 25;18(5):e0286308. doi: 10.1371/journal.pone.0286308 (PMC10212163; doi:10.1371/journal.pone.0286308)
Supplement: S2 Table — (DOCX) [file pone.0286308.s002.docx]

S2 Table: Descriptions of the 48 original qualitative research articles included in the review

| **First Author, Title, Year** | **Cancer type(s)** | **Country** | **Number of patient participants** | **Study population** | **Scope of study** | **Data collection method** |
| --- | --- | --- | --- | --- | --- | --- |
| Ahlstedt Karlsson, “It’s not just any pill” – women’s experience of endocrine therapy after breast cancer surgery, 2019 | Breast | Sweden | 25 | Female surgical outpatients. Women diagnosed with breast cancer and treated with ET (i.e., Tamoxifen) after surgery. | To explore women’s experiences of taking endocrine therapy after breast cancer surgery, the challenges and strategies used to manage endocrine therapy | Focus groups |
| Allen, The transition from breast cancer “patient” to “survivor”, 2009 | Breast | USA | 47 | Females who had stage one or two breast cancer and had completed adjuvant chemotherapy or radiation therapy within the last 12 months | To explore women’s fears regarding risk of recurrence, sense of loss of medical monitoring and social support, and the strategies used to cope with these issues | Focus groups |
| Anderson, Lifestyle issues for colorectal cancer survivors – perceived needs, beliefs and opportunities, 2013 | Colorectal | UK | 40 | “Survivors” of colorectal cancer who have completed treatment | To explore perceived need for advice on diet, activity, and beliefs about the role of lifestyle for reducing disease recurrence | Focus groups |
| Appleton, Patients’ experiences of living beyond colorectal cancer. A qualitative study, 2013 | Colorectal | UK | 13 | Individuals who had completed curative treatment from the time of completion of treatment to five years later | To explore experiences in the period following cancer treatment and the physical, psychological, and social aspects associated with adjusting to everyday life | Interviews |
| Ashing-Giwa, Understanding the breast cancer experience of women: a qualitative study of African American, Asian American, Latina and Caucasian cancer survivors, 2004 | Breast | USA | 102 | Females, multi-ethnic population of women diagnosed with all stages of breast cancer | To investigate the psychosocial effects, challenges, and concerns of a multi-ethnic population of women with breast cancer | Focus groups; also conducted interviews with 20 ‘key informants’ (not patients) |
| Barlow, “Abandoned by medicine”? A qualitative study of women’s experiences with lymphoedema secondary to cancer, and the implications for care, 2014 | Breast (and other cancers causing lymphoedema) | UK | 133 (15 interviews, 14 in FGs, plus data from 104 respondents to a postal questionnaire) | Individuals diagnosed with a cancer causing lymphoedema between two and 30 years since diagnosis | To explore experiences of lymphoedema and the “journey” from diagnosis, to treatment and maintenance of the condition | Interviews, focus groups, and free text questionnaire responses |
| Boykoff, Confronting chemobrain: an in-depth look at survivors’ reports of impact on work, social networks, and health care response, 2009 | Breast | USA | 74 (20 participated in initial FGs then all 74 participated in individual interviews) | White and African American women who had completed radiation or chemotherapy at least a year ago | To understand the psychosocial impact of chemobrain on women’s personal and professional lives | In-depth interviews and focus groups |
| Cahir, Women’s experiences of hormonal therapy for breast cancer: exploring influences on medication-taking behaviour, 2015 | Breast | Ireland | 31 | Women with stage 1-3 breast cancer prescribed adjuvant hormonal therapy | To investigate modifiable influences on adjuvant hormonal therapy medication taking behaviour in women with stage 1-3 breast cancers | Semi-structured interviews |
| Cheng, A qualitative insight into the self-management experience among Chinese breast cancer survivors, 2016 | Breast | China | 19 | Women with breast cancer within 5 years of diagnosis | To explore Chinese breast cancer survivors’ views and experiences of self-management | Analysis of archived interview transcripts |
| Desnoo, A qualitative study of anterior resection syndrome: the experiences of cancer survivors who have undergone resection surgery, 2006 | Rectal | UK | 7 | Individuals who had anterior resection surgery in the past and had stoma reversal at least 6 months prior to study starting, without disease progression | To explore how individuals recovered and adapted following surgical resection of their rectal cancer and the syndrome that occurs as a consequence of this operation | Semi-structured interviews |
| El-Turk, Treatment burden experienced by patients with lung cancer, 2021 | Lung | Australia | 16 | Individuals attending a tertiary hospital cancer centre who were undergoing or had completed ablative therapy, chemotherapy, or immunotherapy for lung cancer within the past six months | To understand treatment burden after lung cancer | Semi-structured interviews |
| Fu, Breast Cancer Survivors’ Intentions of Managing Lymphedema, 2005 | Breast | USA | 12 | Breast cancer survivors who completed surgical treatment at least 3 months before enrolling in study, and having diagnosis of lymphedema for at least 1 month prior to enrolling in study | To describe breast cancer survivors’ experiences of managing lymphedema in their daily lives. | Semi-structured interviews |
| Goodwin, Older adults’ functional performance loss and adaptation during chemotherapy, 2007 | Lung, breast, colon, and pancreas | USA | 11 | Individuals attending an oncology centre within a teaching hospital who were aged 65 or older, and undergoing chemotherapy with at least two months of chemotherapy already completed | To describe changes in functional performance in older adults during chemotherapy | Semi-structured interviews |
| Greenslade, Living with lymphedema: a qualitative study of women’s perspectives on prevention and management following breast cancer-related treatment, 2006 | Breast | Canada | 13 | Women with lymphedema of the upper extremity for at least a year and with no evidence of active cancer | To investigate women’s experiences of living with lymphedema | Semi-structured interviews |
| Hall, Perceptions of time spent pursuing cancer care among patients, caregivers, and oncology professionals, 2021 | Lung and melanoma | USA | 11 | Individuals with a diagnosis of stage 3 or 4 melanoma or lung cancer, receiving active cancer treatment | To explore perceptions of the time spent receiving cancer care | Semi-structured interviews |
| Hamilton, The impact and management of cancer-related fatigue on patients and families, 2001 | Lung cancer | Canada | 22 | Individuals with primary non-small cell lung cancer without distant metastases, and who were receiving radiation to their thorax only, at six weeks post completion of radiation therapy | To examine the impact of fatigue on individuals with lung cancer and their families and to explore how fatigue was managed | Semi-structured interviews |
| Hiltrop, Conflicting demands, coping, and adjustment: a grounded theory to understand rehabilitation processes in long-term breast cancer survivors, 2021 | Breast | Germany | 26 | ‘Survivors’ 5-6 years after diagnosis | To explore rehabilitation processes in cancer survivors | Semi-structured interviews |
| Ho, A qualitative focus group study to identify the needs of survivors of stage II and III colorectal cancer, 2016 | Colorectal cancer | Canada | 30 | “Survivors” who had completed their primary active anti-cancer treatments | To identify the concerns of colorectal cancer survivors in terms of physical functioning, psychological wellbeing and social relationships | Focus groups |
| Şengün İnan, Experiences of Turkish survivors of breast cancer: Neuman systems model perspective, 2016 | Breast | Turkey | 14 | “Survivors” who had completed their primary treatment of breast cancer at least 3 months ago and up to 3 years after treatment | To describe the post-treatment experiences of Turkish survivors of breast cancer (using the Neuman systems model). | Semi-structured in-depth interviews |
| Jakobsson, The lived experience of recovery during the first 6 months after colorectal cancer surgery, 2017 | Colorectal | Sweden | 10 | Individuals attending a surgical unit for elective colorectal surgery at a University hospital at one and six months post surgery | To describe lived experiences of recovery during the first 6 months of colorectal cancer surgery | Semi-structured interviews |
| Jeffs, Exploring patient perception of success and benefit in self-management of breast-cancer related arm lymphedema, 2016 | Breast | UK | 21 | Men and women attending a lymphoedema service and engaged in breast cancer related lymphedema for at least six months | To explore perceptions of success and benefit with self-management of breast cancer-related lymphedema and to explore how patients decide whether their swollen limb has improved or deteriorated | In-depth interviews |
| Kidd, Experiences of self-care in patients with colorectal cancer: a longitudinal study, 2008 | Colorectal | UK | 11 | Individuals undergoing chemotherapy at a hospital cancer centre, interviewed at the start and end of a chemotherapy | To explore patients’ experiences of self-care during a six month course of chemotherapy treatment for colorectal cancer | Semi-structured interviews |
| Kuo, Symptom management tasks and behaviours related to chemotherapy in Taiwanese outpatients with breast cancer, 2015 | Breast | Taiwan | 17 | Individuals who had completed chemotherapy for breast cancer and were attending an outpatient clinic | To explore self-management behaviours, tasks and challenges experienced by individuals experiencing chemotherapy symptoms | Semi-structured interviews |
| Liamputtong, Breast cancer diagnosis: biographical disruption, emotional experiences, and strategic management in Thai women with breast cancer, 2015 | Breast | Thailand | 20 | Women who had commenced or completed treatment for breast cancer | To understand the meanings and emotional burdens experienced by women with breast cancer in southern Thailand | In-depth interviewing and drawing methods |
| Lindquist, Self-care among female cancer survivors with secondary lymphedema: a qualitative study, 2015 | Breast, gynaecological, and melanoma | Sweden | 8 | Women attending a rehabilitation clinic | To describe the experiences of self-management of lymphedema | Individual open interviews |
| Lo, Health self-management experiences of colorectal cancer patients in postoperative recovery: a qualitative study, 2021 | Colorectal | Taiwan | 10 | Individuals attending a surgical outpatient clinic and within one month of colorectal cancer resection | To explore self-management experiences and post-discharge support needs after colorectal cancer surgery | Semi-structured interviews |
| Lu, The lived experiences of patients with post-operative rectal cancer who suffer from altered bowel function: A phenomenological study, 2017 | Rectal | Taiwan | 16 | Individuals diagnosed with adenocarcinoma in the recto-sigmoid junction or rectum, and treated with either sphincter-saving surgery alone or with temporary stoma reversal and suffered at least one symptom of post-operative bowel function | To explore the lived experiences of post-operative rectal cancer patients with altered bowel function. | In-depth semi-structured interviews |
| McMullen, The Greatest Challenges Reported by Long-Term Colorectal Cancer Survivors with Stomas, 2008 | Colorectal | USA | 178 | Colon and rectal cancer survivors with permanent ostomies diagnosed between 2000-2006 | To assess the greatest challenges of living with ostomies and identify key issues for teaching and intervention. | Open-ended survey questions |
| Milne, Men’s experiences following laparoscopic radical prostatectomy: A qualitative descriptive study, 2008 | Prostate | Canada | 19 (5 individual interviews and 3 focus groups) | Men who had undergone LRP during the previous 3-year period | To explore what men experience following laparoscopic radical prostatectomy and how adequately their pre- and postoperative needs are being met. | Interviews and focus groups |
| Ose, Complexity of care and strategies of self-management in patients with colorectal cancer, 2017 | Colorectal | Germany | 12 | Patients diagnosed with colorectal cancer | To analyse and describe the complexity of individual patient situations and to analyse and describe already established  self-management strategies of patients to handle this complexity. | Focus groups |
| Prabandari, “Alas, my sickness has become my family’s burden”: A nested qualitative study on the experience of advanced breast cancer patients across the disease trajectory in Indonesia, 2022 | Breast | Indonesia | 20 | Individuals with metastatic breast cancer | To explore the narratives of patients with metastatic breast cancer to understand their experiences from diagnosis to accessing and undergoing cancer treatments. | Semi-structured interviews |
| Reinwalds, A Descriptive, Qualitative Study to Assess Patient Experiences Following Stoma Reversal After Rectal Cancer Surgery, 2017 | Rectal | Sweden | 16 | Individuals who had been operated for rectal cancer with an anterior resection and a temporary loop ileostomy that had been reversed | To describe the patient experience during the first 4 to 6 weeks after reversal of a temporary loop ileostomy due to rectal cancer. | Interviews |
| Ridner, Breast cancer survivors’ perspectives of critical lymphedema self-care support needs, 2016 | Breast | USA | 21 | Individuals with stage II lymphoedema subsequent to breast cancer treatment | To investigate breast cancer survivors’ perspectives on the issues they face related to lymphedema self-care and identify perceived support needs. | Focus groups |
| Saunders, A qualitative study exploring what it takes to be physically active with a stoma after surgery for rectal cancer, 2019 | Rectal | Canada | 15 | Individuals who had been diagnosed with non-metastatic rectal cancer, had undergone surgery for the placement of a stoma, and had completed chemotherapy. | To gain insight into the experiences of rectal cancer survivors who were living with a stoma and understand the impact on their engagement in physical activity. | Semi-structured interviews |
| Schulman-Green, Self-management and transitions in women with advanced breast cancer, 2011 | Breast | USA | 15 | Women who had a diagnosis of metastatic breast cancer, had failed first-line therapy for metastatic disease, and were undergoing any type of treatment. | To describe experiences of self-management in the context of transitions among women with advanced breast cancer. | Semi-structured interviews |
| Shih, Lived Experience among Patients Newly Diagnosed with Lung Adenocarcinoma Stage IV within One Year, 2015 | Lung | Taiwan | 12 | Individuals diagnosed with lung adenocarcinoma stage IV | To explore lived experience among patients newly diagnosed with lung adenocarcinoma stage IV within one year. | Semi-structured interviews |
| Sleight, Occupational Engagement in Low-Income Latina Breast Cancer Survivors, 2017 | Breast | USA | 9 | Individuals who self-identified as Latina, had a diagnosis of breast cancer, and had completed primary surgical treatment, chemotherapy, or radiation | To fill a gap in the literature by exploring the interplay among occupational engagement, socioeconomic status, culture, and QOL in low-income Latina breast cancer survivors. | Semi-structured interviews |
| Sun, The influence of breast cancer related lymphedema on women’s return to work, 2020 | Breast | USA | 13 | Breast cancer survivors more than 12 months post-surgery and radiation treatment, were subsequently diagnosed with lymphedema, and were employed or self-employed at time of developing lymphedema | To address this gap by investigating survivors’ perspectives regarding the ways in which BCRL influences their work and by examining the contextual facilitators and barriers as survivors return-to-work | Semi-structured interviews |
| Thomsen, Patients’ Vulnerability in Follow-up After Colorectal Cancer: A Qualitative Action Research Study, 2017 | Colorectal | Denmark | 12 | Individuals who had undergone fast-track surgery for colorectal cancer and had been discharged from hospital within expected time (at 1 and 3 months after discharge) | To identify the perspectives of fast-track colorectal cancer surgery patients on challenges experienced in the transition from being a hospitalized patient with cancer to being a cancer survivor | Semi-structured interviews |
| Timmons, "It's at a Time in Your Life When You Are Most Vulnerable": A Qualitative Exploration of the Financial Impact of a Cancer Diagnosis and Implications for Financial Protection in Health, 2013 | Breast, lung, and prostate | Ireland | 20 | Primary breast, prostate, or lung cancer patients who were post-initial treatment and had reported they were experiencing ‘extra costs’ or ‘financial difficulties’ because of their cancer to a health professional | To explore:  1. the financial adjustments needed by patients to cope with their financial situation after a cancer diagnosis and  2. the impact of these on financial distress or well-being. | Semi-structured interviews |
| Vrkljan, Meaning of occupational engagement in life-threatening illness: A qualitative pilot project, 2001 | Breast | Canada | 3 | Patients who had completed active treatment at least six months prior to first interview | To explore occupational engagement and its meaning to individuals following a life threatening diagnosis. | Semi-structured interviews |
| Walshe, Coping Well with Advanced Cancer: A Serial Qualitative Interview Study with Patients and Family Carers, 2017 | Breast, prostate, lung, and colorectal | UK | 26 (all 26 in individual interview 1, 19 in individual interview 2, and 16 in 4 focus groups) | Patients with advanced (stage 3-4) breast, prostate, lung, or colorectal cancer, or in receipt of palliative care | To understand successful strategies used by people to cope well when living with advanced cancer; to explore how professionals can support effective coping strategies; to understand how to support development of effective coping strategies for patients and family carers | Serial interviews and focus groups |
| Webber, Breakthrough pain: a qualitative study involving patients with advanced cancer, 2011 | Breast, colorectal, rectal, prostate, lung (as well as ovarian, bladder, oesophageal) | UK | 10 | Individuals with a diagnosis of cancer and a diagnosis of breakthrough cancer pain | To explore the individual experience of living with breakthrough cancer pain using a qualitative methodology | Semi-structured interviews |
| White, Decision-Making Control: Why Men Decline Treatment for Prostate Cancer, 2003 | Prostate | Canada | 31 | Patients with prostate cancer | To conduct a qualitative analysis of decision-making control by men with prostate cancer who refuse conventional cancer therapies. | Re-analysis of transcripts from larger qualitative study |
| Wickersham, Surviving with lung cancer: Medication-taking and oral targeted therapy, 2014 | Lung | USA | 13 | Individuals with Non Small Cell Lung Cancer (any type/stage) receiving an oral epidermal growth factor receptor inhibitors. | To explore the process of medication-taking for adults with non small cell lung cancer taking oral epidermal growth factor receptor inhibitors. | In-depth semi-formal interviews |
| Williams, A phenomenological study of the lived experiences of people with lymphoedema, 2004 | Breast (as well as cervical, carcinoma of the penis, and other non-cancer related lymphedema) | UK | 15 | Individuals with a diagnosis of lymphedema over 3 months duration | To describe the experiences of people living with lymphoedema and explore individuals’ experiences of lymphoedema treatment | In-depth interviews |
| Wu, Cancer-related fatigue: “It’s so much more than just being tired”, 2007 | Breast (and large cell non-Hodgkin’s lymphoma) | USA | 10 (10 interviews, 6 of which also completed diary) | People with cancer who self-reported fatigue | To explore the meaning of cancer-related fatigue from the patients’ experience and perspective. | Semi-structured interviews and 2-week diary |
| Zhang, The meaning of life according to patients with advanced lung cancer: a qualitative study, 2022 | Lung | China | 21 | Individuals with advanced (stage 3 or 4) lung cancer | To explore the meaning of life and end-of-life coping strategies among patients in China with advanced lung cancer. | Semi-structured interviews |
