# Supplementary material for: Treatment burden in individuals living with and beyond cancer: A systematic review of qualitative literature
Source: PLoS One. 2023 May 25;18(5):e0286308. doi: 10.1371/journal.pone.0286308 (PMC10212163; doi:10.1371/journal.pone.0286308)
Supplement: S1 Appendix — (DOCX) [file pone.0286308.s004.docx]

**S1 Appendix.** Medline search strategy.

1. exp Colonic Neoplasms/

2. bowel cancer.tw.

3. bowel neoplasm?.tw.

4. bowel tumo?r?.tw.

5. colon$ tumo?r?.tw.

6. colon$ cancer$.tw.

7. colorectal cancer?.tw.

8. colorectal neoplasm?.tw.

9. colorectal tumo?r?.tw.

10. exp Rectal Neoplasms/

11. rectal cancer?.tw.

12. exp Prostatic Neoplasms/

13. prostate cancer?.tw.

14. prostate neoplasms.tw.

15. prostatic cancer.tw.

16. exp Breast Neoplasms/

17. breast cancer?.tw.

18. breast neoplasms.tw.

19. breast cancer.tw.

20. exp Lung Neoplasms/

21. lung cancer?.tw.

22. Bronchial Neoplasms/

23. lung neoplasms.tw.

24. lung cancer.mp.

25. 1 or 2 or 3 or 4 or 5 or 6 or 7 or 8 or 9 or 10 or 11 or 12 or 13 or 14 or 15 or 16 or 17 or 18 or 19 or 20 or 21 or 22 or 23 or 24

26. treatment burden$.tw.

27. burden$ of treatment$.tw.

28. (burden$ adj2 (treat$ or therap$)).tw.

29. (burden$ adj2 (treat$ or therap$)).tw.

30. Fatigue/

31. Cancer Pain/ or Pain/

32. exp Affect/

33. Stress, Psychological/

34. "Activities of Daily Living"/

35. Antineoplastic Agents/ae [Adverse Effects]

36. Antineoplastic Combined Chemotherapy Protocols/ae [Adverse Effects]

37. Radiotherapy/ae [Adverse Effects]

38. Radiotherapy, Adjuvant/ae [Adverse Effects]

39. Surgical Stomas/

40. Life Change Events/

41. (cancer$ adj3 surger$).tw.

42. health behavior/ or "treatment adherence and compliance"/ or "patient acceptance of health care"/ or treatment refusal/

43. emotional adjustment/ or survivorship/

44. Self Care/

45. Medication Adherence/

46. Rehabilitation/

47. (treatment adj4 (regimen or regime or task? or work)).tw.

48. social support/ or exp family/

49. Exercise/

50. well being.tw.

51. diet/

52. lifestyle/

53. appointment*.mp.

54. 26 or 27 or 28 or 29 or 30 or 31 or 32 or 33 or 34 or 35 or 36 or 37 or 38 or 39 or 40 or 41 or 42 or 43 or 44 or 45 or 46 or 47 or 48 or 49 or 50 or 51 or 52 or 53

55. (qualitative or ethnograph* or phenomenol* or ethnonurs* or grounded theor* or purposive sample or hermeneutic* or heuristic* or semiotics or lived experience* or narrative* or life experiences or cluster sample or action research or observational method or content analysis or thematic analysis or constant comparative method or field stud* or theoretical sample or discourse analysis or focus group* or ethnological research or ethnomethodolog*).mp. or interview*.tw. [mp=title, abstract, original title, name of substance word, subject heading word, floating sub-heading word, keyword heading word, organism supplementary concept word, protocol supplementary concept word, rare disease supplementary concept word, unique identifier, synonyms]

56. 25 and 54 and 55

57. limit 56 to (english language and humans and yr="2001 -Current")

58. cancer screening.tw.

59. 57 not 58

60. exp "review"/

61. 59 not 60
