# Supplementary material for: Treatment burden in individuals living with and beyond cancer: A systematic review of qualitative literature
Source: PLoS One. 2023 May 25;18(5):e0286308. doi: 10.1371/journal.pone.0286308 (PMC10212163; doi:10.1371/journal.pone.0286308)
Supplement: Attachment — Submitted filename: Response to reviewers_180423.docx [file pone.0286308.s006.docx]

**Academic Primary Care**

School of Medicine, Dentistry and Nutrition

Division of Applied Health Sciences

Polwarth Building

Foresterhill

Aberdeen AB25 2ZD

Scotland

United Kingdom

Tel: +44 (0) 1224 437264

Email: primarycare@abdn.ac.uk

**Editorial Team, PLOS One**

**Manuscript** PONE-D-22-21252R1

**Title: Treatment burden in individuals living with and beyond cancer: a systematic review of qualitative literature**

**POINT BY POINT RESPONSES TO REVIEWER COMMENTS**

**REVIEWER THREE**

COMMENT: The article is well written and it addresses an important topic. I do not see any outstanding issues as regarding the methodology of the study and the article structure.

RESPONSE: Thank you and we are pleased that the manuscript is almost ready for publication.

COMMENT: I do not completely agree with the term "work" used by the authors throughout the manuscript. Specifically in some cases as for example at page 17 I would substitute "Work was time-consuming and included cognitive work, practical work, and relational work." with "the cancer experience was time-consuming and included cognitive work, practical work, and relational work." This appears also at page 23 in "The influence of cancer type and stage chapter", where "Severe and persistent symptoms could make health care work more difficult". The literature talks extensively of the difficult self-management and burden of cancer patients and in line with the literature I would change the term "work" with "management".

RESPONSE: We have reviewed the article and made changes to the term “work” throughout. We made the following changes:

Results, Theme one: changed “*Work was time consuming*” to “*The cancer experience was time-consuming*”.

Results, Theme two: deleted “*The work of managing cancer took place in this context*”.

Results Theme three: changed “*In order to undertake the work of cancer management, individuals had to draw on personal and healthcare resources*” to “*Individuals used personal and healthcare system resources to manage cancer*.”

Results, Theme four: changed “*Individuals were burdened by health care work when there was a mismatch*…” to “*Individuals were burdened by health care when there was a mismatch…”*

Results, the influence of cancer type and stage, changed “*Severe and persistent symptoms could make health care work more difficult*” to “*Severe and persistent symptoms contributed to treatment burden.*”

Discussion, main findings, changed “The work of cancer management” to “Cancer management”, and “this same work can become burdensome” to “treatment burden occurs when…”

Discussion, comparison with existing literature: changed “*cancer-related work*” to “*cancer related health management*”, and “*self-management work*” to “*self-management activities*”.

COMMENT: Discussion - main findings:
The notion of abandonment and shift in responsibility is not discussed taking into account the figure of the nurse case manager, which exists in certain healthcare systems and can help to smooth the transition at the end of cancer treatment, in the new phase of the disease trajectory survivorship.

RESPONSE: We have added the following paragraph, which introduces the concept of the nurse care manager. Three additional references have been added which relate to patient navigation, care managers, and evaluation of these roles in cancer.

*“A key finding in this review is that consumption of patients’ time can lead to treatment burden. There is increasing recognition of “time toxicity” in cancer care, which is conceptualised as time spent coordinating care and in frequent visits to healthcare facilities [91].*

*Some healthcare systems have introduced patient navigation and nurse case manager roles in which a dedicated person or team helps patients to navigate complex systems, coordinates care, and helps patients to manage transitions in care [92]. Navigators and case managers can improve patient satisfaction and measures of patient experience with their care [92, 93]. Evidence about their effectiveness on reducing hospitalisation, use of emergency services and overall cost effectiveness is less clear [92,94]. This review suggests that coordinating care and visiting healthcare facilities form only a small part of the work of managing cancer. Patients’ time is consumed by cognitive, relational, and other practical processes related to cancer management. Many of these processes take place away from healthcare settings.”*
